# Digital Twin-Driven Optimization of Pilot-Scale Polyurethane Aerogel Production Using SVR Modelling

**DOI:** 10.3390/gels12060483

**Published:** 2026-06-01

**Authors:** Óscar Brandón-Basdediós, Laura Miguélez-Riádigos, Esther Pinilla-Peñalver, Mateo Alonso, Paula Sánchez, Luz Sánchez-Silva, Juan Luis Sobreira-Seoane

**Affiliations:** 1 Instituto Tecnológico de Galicia (ITG), Cantón Grande 9, Planta 3, 15003 A Coruña, Spain; 2 Department of Chemical Engineering, University of Castilla-La Mancha, Avda. Camilo José Cela 12, 13071 Ciudad Real, Spain

**Keywords:** polyurethane aerogels, digital twins, sustainable manufacturing, data-driven modelling

## Abstract

The growing demand for sustainable and energy-efficient materials has positioned aerogels as promising candidates for advanced insulation applications. Among them, polyurethane (PU) aerogels are attracting increasing interest due to their thermal insulation properties and mechanical versatility. However, their development commonly relies on trial-and-error experimentation, which is time-consuming and resource-intensive. This study presents a Digital Twin (DT) framework to support PU aerogel design and reduce the experimental workload. A pilot-scale DT was developed using data from 21 synthesis experiments, including process configuration, parameter mapping, model development, and process analysis. Two predictive models were evaluated, with the Support Vector Regression (SVR) model showing good agreement with the experimental data (R^2^ = 0.964) and being selected to estimate aerogel density within the parameter range studied. The DT framework enabled the identification of synthesis conditions associated with lower density, which may contribute to improved thermal insulation performance. These results illustrate the potential of DT-assisted modelling to support material development, improve process understanding, and guide more efficient experimentation in PU aerogel synthesis. Overall, this work highlights a data-driven approach for advancing sustainable and scalable aerogel manufacturing.

## 1. Introduction

Polymeric materials are widely valued for their excellent physicochemical properties and versatile applications. Among them, aerogels stand out as a unique class of ultralight weight and highly porous materials characterized by low density, high specific surface area, and exceptional thermal insulation properties [1,2]. Their extraordinary thermal performance is fundamentally governed by their nanostructured framework, where a highly interconnected mesoporous network restricts the mean free path of gas molecules via the Knudsen effect, effectively minimizing gaseous thermal conduction [3]. While traditional silica aerogels are well known for these properties, their inherent brittleness and structural fragility severely limit their practical handling and widespread applicability [1,4]. Consequently, polymer-based aerogels, particularly polyurethane (PU) aerogels, have emerged as a robust alternative. PU aerogels uniquely combine the intrinsic thermal properties of conventional aerogels with superior mechanical integrity, tailored flexibility, and versatile macromolecular chemistry, enabling their use in demanding sectors such as energy-efficient construction, aerospace, environmental protection, and biomedical applications [5].

Despite their outstanding potential, the industrial implementation of PU aerogels remains constrained by high production costs and the significant complexities associated with scaling up from the laboratory to the pilot scale. The synthesis of these advanced materials relies on highly sensitive multistep procedures, typically involving sol–gel polymerization, structural aging, and delicate drying phases, where minor deviations in process parameters can lead to pore collapse and microstructural degradation. Addressing these limitations is essential to fully exploit their potential and support sustainable innovation. In this context, freeze-drying has emerged as a promising processing route for aerogel fabrication [1,6]. By driving the phase transition of the frozen solvent through sublimation, freeze-drying mitigates the destructive capillary forces typically present at the liquid–vapor interface. This approach offers improved control over the final morpho-structural properties, avoids the severe operational conditions of traditional supercritical drying, and facilitates their transition toward industrial-scale production.

To further support scalable production, predictive models play an important role in addressing key challenges in material development, particularly in aerogel manufacturing. These models reduce reliance on extensive trial-and-error experimentation in pilot-scale processes by leveraging experimental data and machine learning (ML) approaches. In doing so, they enable the prediction of critical properties such as density and thermal conductivity, thus enabling faster material optimization and scale-up processes. This data-driven approach improves efficiency and reduces both development time and costs, and supports the design of advanced materials with tailored properties, thereby contributing to the sustainable industrial integration of aerogels.

In this context, recent studies have shown that machine learning models can capture complex relationships governing aerogel synthesis and performance. Tafreshi et al. [7] presented one of the first frameworks for predicting multiple properties of nanostructured aerogels, using artificial neural networks to estimate density, porosity, and compressive modulus of polyimide aerogels from synthesis descriptors. Subsequent studies extended this approach to thermal performance, applying supervised learning methods to estimate the thermal conductivity of polymeric aerogels [8].

More recently, ensemble learning techniques have been used to predict both mechanical and thermal properties in aerogel-based composites [9], while advanced models have also been applied to predict the cooling performance of radiative cooling aerogels by combining material composition, optical properties and environmental parameters [10]. Alongside these developments, literature has increasingly pointed to hybrid modelling strategies that combine data-driven methods with modelling and simulation as a way to address some of the limitations of purely data-driven approaches [11].

Nonetheless, achieving precise control over the development of advanced materials such as aerogels remains challenging due to the limitations of traditional experimental and simulation approaches. These methods are often associated with long development cycles, high operational costs, and simplified representations of complex coupled physical phenomena, particularly during drying processes. As a result, the prediction of final material properties may be limited in accuracy, highlighting the need for more advanced data-driven and model-based tools to better capture process–structure–property relationships.

In this context, Digital Twins (DTs) offer a powerful framework for creating virtual representations of real-world systems to support material property optimization, production processes monitoring, system behaviour prediction, and efficient resource allocation [12,13]. Initially developed and widely applied in sectors such as aerospace and energy [14], DTs have more recently gained increasing attention in biomanufacturing and materials engineering. They are a key component of the Industry 4.0 paradigm, where they are integrated with enabling technologies such as the Industrial Internet of Things (IIoT), Machine Learning (ML), Artificial Intelligence (AI), and cloud computing to enhance process efficiency and product quality. In this context, DTs support data-driven quality management approaches based on frameworks such as Quality by Design (QbD) and Quality by Digital Design (QbDD) [15].

Successful implementation of DTs requires well-defined modelling frameworks. The 3D model proposed by Grieves describes the interactions between the physical system, its virtual counterpart, and the data connections linking both domains. This concept was later extended by Tao into a 5D framework, which incorporates data and service dimensions to better capture the role of heterogeneous data sources and platform-based services for simulation, monitoring, and optimization tasks [16]. These extended architectures provide a structured foundation for integrating complex process data, such as those generated in aerogel manufacturing.

DTs have already demonstrated their versatility across various materials, showcasing their potential for quality prediction, process optimization, and design enhancement. For composite materials, Wang et al. [17] developed a visual DT model to study the curing process, while Fernández et al. [18] created a DT to detect resin flow irregularities, such as dry spots and areas with inadequate impregnation during resin transfer molding. For thermoplastic composites, DTs have been utilized for real-time monitoring and predictive analysis [19], with Hürkamp et al. [20] designing a simulation-based DT for thermoforming processes to aid in design and inline quality control, as well as for predicting interface bond strength based on process settings. In flexible materials, DTs have enabled real-time adaptation by integrating control and planning systems [21], while Jaboviste et al. [22] employed a DT for viscoelastic materials to study the evolution of storage modulus under strain, addressing a complex nonlinear phenomenon. For polyurethane (PU), Stanko and Stommel developed a DT for rotational molding, optimizing part design, mold configuration, and process parameters [23].

Together, these examples show that DTs are particularly effective for complex material systems where strong coupling exists between process conditions and final material properties. However, despite this broad range of applications, the transfer of DT concepts to aerogel production has received comparatively limited attention. In the context of aerogels, DT applications and AI-driven methodologies are an emerging frontier, although current research remains fragmented. Recent studies have predominantly focused on microstructural modeling; for instance, Lebedev et al. developed a DT framework to simulate the porous architecture of chitosan-based aerogels using cellular automation, focusing on property prediction and partial replacement of experimental efforts [24].

Other authors have successfully applied ML to specific domains: Walker et al. [25] established synthesis-property predictions specifically for silica aerogels, while Hosseinpoor et al. [26] employed AI to optimize the adsorption performance of modified carbon aerogels. Furthermore, ML has been used to evaluate the energy performance of aerogel-based glazing systems in construction [27].

Despite these advancements, a significant gap persists regarding a holistic DT framework that integrates pilot-scale synthesis parameters with a structured deployment methodology. This study addresses this gap by proposing a cloud-based DT environment for PU aerogel production at a pilot scale. Crucially, to move beyond isolated algorithmic predictions and establish a clear methodological linkage with lifecycle architectures, our implementation is explicitly structured around three core dimensions: standardized data management across production stages, tool accessibility for materials experts, and knowledge integration through Quality by Design (QbD) principles. By embedding physical boundaries into a model, the proposed framework ensures that the predictive system prioritizes solutions that are both structurally viable and optimized for thermal insulation performance. The subsequent sections detail the deployment and specific components of this lifecycle-driven digital twin framework.

## 2. Results and Discussion

### 2.1. Density and Thermal Conductivity Results

A diverse range of aerogel samples was synthesized by varying key parameters, including the organic solvent, solids content, and molecular weight of the polyol. These modifications aimed to optimize density and thermal conductivity while navigating several phenomenological trade-offs that govern the material’s integrity. The results are summarized in Table 1.

Regarding chemical stability, a critical threshold was identified at ethylenediamine/hexamethylene diisocyanate (EDA/HMDI) ratios exceeding 0.25. In this regime, the presence of excess primary amines in the polymer matrix triggers oxidative degradation and the formation of chromophores [28], leading to the characteristic yellowing and structural embrittlement of the aerogels. Furthermore, these samples exhibited reduced coating thickness and significant shrinkage, compromising their long-term stability and suitability for practical applications, as previously detailed in [29]. Conversely, excessively low ratios are equally detrimental, as they hinder the complete conversion of the initial isocyanate groups, leading to incomplete polymerization and an under-crosslinked network [30].

The impact of the polyol molecular weight was found to be markedly non-linear, presenting a compromise between structural optimization and processing feasibility. On one hand, increasing the PEG molecular weight (within the 600–4000 g/mol range) promotes lower aerogel density. This is physically attributed to a decrease in the effective crosslink density; longer PEG chains increase the spacing between junction points and expand the soft segments, generating a more flexible and open PU network. This enlarged macromolecular free volume reduces the capillary pressure exerted during the drying phase, facilitating solvent removal without the collapse of the mesoporous framework [31].

On the other hand, polyols at the upper limit of this range tend to induce premature agglomeration during polymerization. The increased viscosity and reduced mobility of higher molecular weight chains hinder reaction kinetics [32], ultimately rendering the synthesis unfeasible.

**Figure 1:**
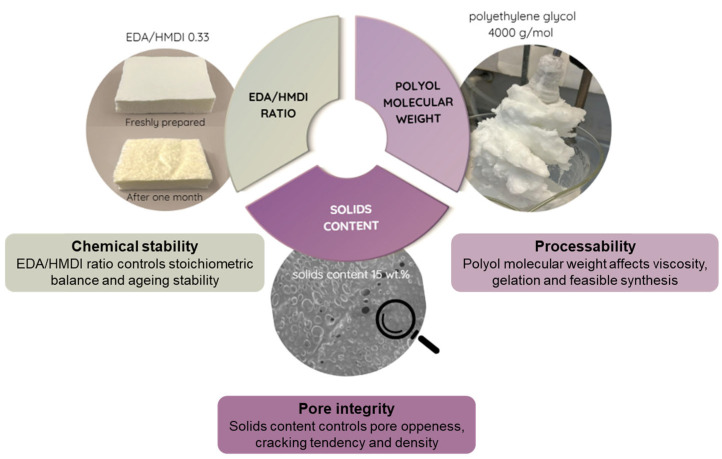
Experimental processability map of PU aerogel synthesis showing the effects of key synthesis variables, including EDA/HMDI ratio, polyol molecular weight, and solids content, on sample stability, density, pore integrity, and synthesis failure.

Additionally, the solids content plays a decisive role in the final morphology. It was observed that solids contents above 12 wt.% promote a transition toward a closed-pore morphology. The higher concentration of polymer per unit increases the internal stress during solvent evaporation. Consequently, these rigid structures are unable to dissipate capillary-induced strain, making the materials highly susceptible to macro-cracking and significantly increasing their density. A visual summary of these synthesis boundaries and the resulting “feasible operational window” is schematized in Figure 1.

Since density is closely related to the thermal insulation performance of aerogels, this parameter also influences the resulting thermal conductivity. However, although lower density generally contributes to reduced thermal conductivity due to the reduced continuity of the solid polymeric network, this relationship is not always linear in freeze-dried systems. The pore morphology and microstructural features generated during freezing and sublimation can also influence heat transfer through the gaseous phase within the porous structure. Consequently, samples with different densities may exhibit similar thermal conductivity values, as observed in previous studies on highly porous aerogels [29,31,33,34,35].

Crucially, these physical insights, specifically the instability thresholds, the non-linear processing limits of the polyol molecular weight, and the morphological transitions driven by solids content, constitute the foundational logic for the modeling framework discussed in the following sections. Rather than treating the DT as a purely statistical correlation tool, these phenomenological constraints were integrated to define the model’s operational envelope. This ensures that the predictive algorithms prioritize formulations that are not only optimized for thermal performance but are also structurally viable and chemically stable.

### 2.2. Digital Twin as a Driver for Pilot Plants

The DT enabled a shift from ex-post analysis to inform decision-making by allowing the simulation and optimization of production settings beforehand. Key tools used in this research are briefly introduced, with screenshots provided in the Supplementary Materials for reference.

The model’s forecasting capabilities were explored through contour plots, illustrating how input variable combinations affect outcomes such as density (Figure S3A). Feasible regions (Figure S3B) were also identified to determine optimal operating conditions, such as minimizing density.

Two Key Performance Indicators (KPIs)—density (g/cm^3^) and thermal conductivity (W/m·K)—were tracked in the DT, linking aerogel performance properties with production data (Figure S4). A dashboard featuring KPI thresholds, material ratios, and density comparison charts was developed to streamline data analysis as new information was integrated (Figure S5).

### 2.3. Mathematical Modelling of the Process Performance

To evaluate the capability of the models considered for integration into the DT, an evaluation was conducted using the available dataset. The objective was to identify the modelling approach best suited to capture the relationships between input parameters and target properties of the aerogel. Within this evaluation, two types of models were considered: linear models based on OLS Regression and Support Vector Regression with a radial basis function. The subsequent sections present a summary of the results for each approach, followed by a comparative analysis and final model selection. The models for predicting thermophysical properties were developed using the dataset presented in Table S1. In this model, HMDI, triethylamine (TEA), and dimethylol propionic acid (DMPA) are held constant, allowing the focus to shift toward analysing the influence of solids content, the EDA/HMDI ratio, the molecular weight of the selected PEG, and the stirring method employed during dispersion. The experimental dataset comprises 21 runs spanning four input factors, with four replicate pairs (PU-10/PU-13, PU-8/PU-16, PU-11/PU-12 and PU-19/PU-21). The EDA/H12MDI ratio takes five distinct values between 0.09 and 0.33 mol/mol, with most experiments have 0.33 (8 runs) and 0.18 (8 runs) values. Solids content ranges from 2.7 to 10.7 wt.%, with most experiments (15 of 21) clustered between 3.1 and 4.5 wt.% and a few runs sampling the higher end (7.2–10.7 wt.%). PEG molecular weight is sampled at four discrete levels (600, 1000, 2000, and 3350 g/mol), with 2000 g/mol being used for 18 out of 21 runs. The dispersion method includes 15 runs using mechanical agitation and 7 using sonication. The output variables, included in Table 1, span 0.030–0.051 W/m·K for thermal conductivity and 0.034–0.121 g/cm^3^ for density, with both distributions skewed towards the lower end of their respective ranges. This reflects the exploratory nature of the experimental campaign rather than a fully balanced design of experiments, and the predictive scope of the model is consequently strongest in the densely sampled region.

As the dataset is limited, the generalization potential of the model needs to be carefully assessed, opting for simplicity, even at the cost of selecting a model with worse performance metrics. Even with this consideration, the model can be used as a first approximation to guide further experimentation.

After training, two models were selected for analysis. The first is the model that has the potential for least overfitting, which is the Elastic Net regression. The second one is the model that yields the best metrics for predicting the density value, which is the SVR model using 3 principal components.

#### 2.3.1. Elastic Net

The Elastic Net regression applies both types of regularization (L1 and L2) to reduce the value of the coefficients of the linear model to improve generalization. Due to this technique, several factors are deemed not relevant for the model. In Table 2, the value for the coefficient of each considered factor is included.

**Figure 2:**
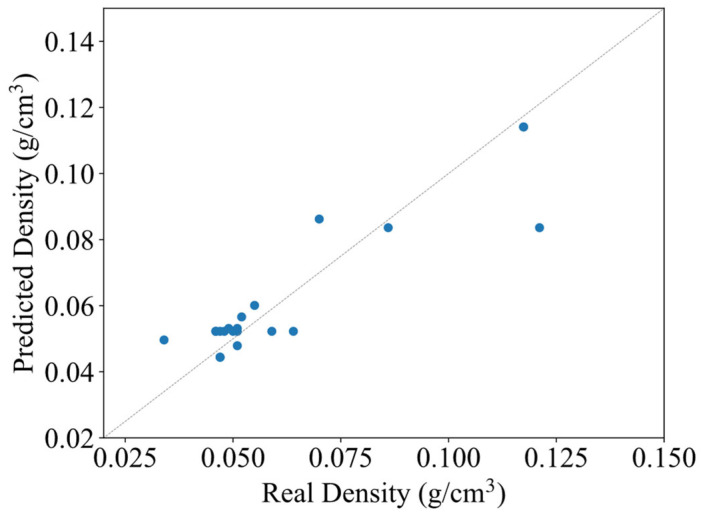
Predicted vs. real density for the developed Elastic Net model. The *x*-axis represents the experimental values, while the *y*-axis shows the model’s predictions for each data point. The grey dotted line represents the ideal case where the prediction equals the real value.

The most relevant input variable is the solids content. To minimize the desired density, the model suggests decreasing the solids content as much as possible. The model demonstrates an acceptable predictive accuracy considering its simplicity, as illustrated in Figure 2, which compares predicted values with experimentally measured data. The results confirm the model’s reliability, with a consistent error margin across the entire experimental range, except for the highest density experiment.

The model fit is further supported by performance metrics. The Elastic Net model achieves an R^2^ of 0.820. Additionally, the model’s error metrics, including MSE (1.2 × 10^4^)^,^ RMSE (1.1 × 10^−2^), and MAE (7.2 × 10^−3^), align with results from similar studies (Table 3). The simplicity of this model is achieved by including the regularization terms to the linear regression model. This makes it appropriate to determine the factors, or factors in this case, that have a larger impact on the final density. However, due to this same reason, it fails to give information on the influence that the rest of the inputs have, and the predicted density may serve as an estimate, but the accuracy of this prediction is limited, owing to the variance present in the dataset for some values of the solids content.

#### 2.3.2. Support Vector Regression

The model that achieved the best performance metrics was the SVR model using a radial basis function as kernel (the remaining hyperparameters are reported in Table 4) and considering all input factors: ‘EDA/H12MDI (mol/mol)’, ‘Solids content calculated (wt.%)’, ‘Sonication (binary)’, and ‘PEG Molecular weight (g/mol)’. As opposed to the Elastic Net model, this model can evaluate the impact of every input variable selected, but, due to this increased model complexity, it has a higher risk of overfitting and not generalizing well to new experiments. Although the dataset consisted of 21 experiments, this size is consistent with that of many experimental studies involving advanced porous materials, where sample generation requires complex multi-step synthesis and characterization procedures. Under these conditions, SVR represents an appropriate choice due to its robustness and good generalization performance in small-data scenarios.

For the SVR model, the hyperparameters tuned via the LOOCV procedure described in Section 4.1.4 were the regularization parameter C, which controls the trade-off between model complexity and training error tolerance, and ε, which defines how much prediction error is acceptable before a penalty is applied. The search ranges were C ∈ [10^−4^, 10^5^] and ε ∈ [10^−10^, 10^−1^], sampled with 10 and 20 log-uniformly spaced values.

**Figure 3:**
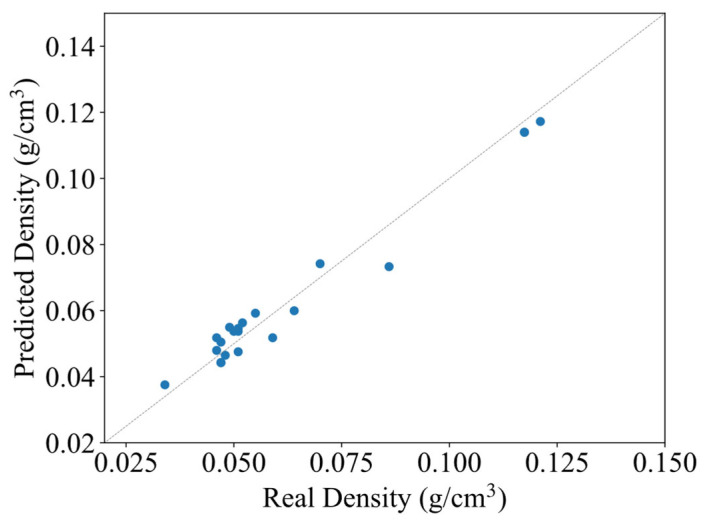
Predicted vs. real density for the developed SVR model. The *x*-axis represents the experimental values, while the *y*-axis shows the model’s predictions for each data point. The grey dotted line represents the ideal case where the prediction equals the real value.

Comparing the predicted values to the measured ones in Figure 3, and the metrics included in Table 3, this model performs better than the Elastic Net on the experiments, as expected, since the radial basis kernel allows for non-linear correlations and the SVR model considers an increased number of input variables. The SVR model achieves an R^2^ of 0.964, an MSE of 2.3 × 10^−5^, an RMSE 4.8 × 10^−3^, and an MAE of 4.2 × 10^−3^.

**Figure 4:**
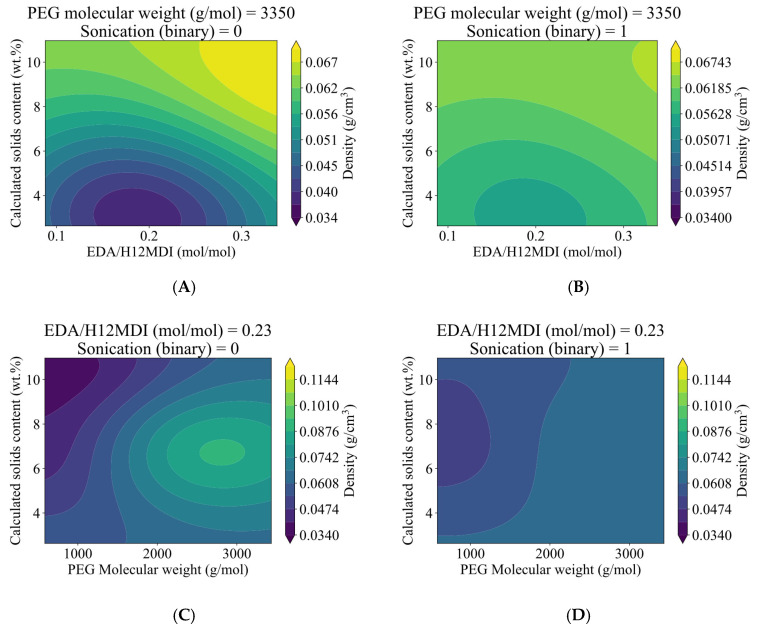
Contour plots of the SVR density model for different parameter values. (**A**,**B**) show the model’s predictions across the experimental ranges of the EDA/H12MDI ratio and solids content, with the PEG molecular weight fixed at its highest value (which yields the minimum predicted density). (**C**,**D**) show the model’s response across different PEG molecular weights and solids content values, with the EDA/H12MDI ratio fixed at the middle of its experimental range. The dispersion method used in (**A**,**C**) is mechanical agitation (corresponding to a ‘Sonication (binary)’ value of 0), while (**B**,**D**) correspond to sonication.

The impact of the studied parameters on density is illustrated in Figure 4, where the contour plots for the model are shown. Figure 4A,B indicates that the optimal EDA/HMDI ratio ranges from 0.15 to 0.25 mol/mol. Regarding PEG molecular mass, Figure 4C shows that higher molecular weights correlate with lower density, with a threshold around 3000 g/mol. This aligns with physical constraints discussed in Section 2.1, where the effects of excess amines or excessively high molecular weight on material stability and processability are addressed.

For calculated solids content, density decreases as this variable decreases, as shown in Figure 4A–C. Furthermore, Figure 4C suggests that the optimal calculated solids content might be lower than the range tested. However, physical limitations in the synthesis process prevent proper aerogel formation if too much water is added during dispersion, as discussed in Section 2.1.

Finally, the dispersion method that results in the lowest density values is mechanical agitation (Sonication =0), as identified when comparing Figure 4A and Figure 4B; and Figure 4C and Figure 4D, respectively.

Taking into account the points discussed above, and the constraints outlined in Section 2.1, an optimal aerogel synthesis point is achieved with a density of 0.037 g/cm^3^. The corresponding experimental parameters for this optimal point are an EDA/HMDI ratio of 0.186 mol/mol, a calculated solids content of 3.25 wt.%, a PEG molecular weight of 3350 g/mol, and mechanical stirring. When comparing this optimal point to the closest experimental results (PU-20 experiment), the relative error in density is 12%. It is worth noting that the reported 12% relative error corresponds to an absolute deviation of only 0.003 g/cm^3^, which is comparable to the experimental variability observed between similar experiments and remains small relative to the full density range explored in the dataset (0.034–0.121 g/cm^3^).

Additionally, the conductivity value for these process conditions is validated, yielding 0.034 W/m·K. This value lies within the lower end of the experimental conductivity range (0.051 to 0.030 W/m·K), suggesting that the model and the developed optimal point effectively minimize the key properties influencing aerogel characteristics.

Although the SVR model achieves performance metrics that compare favourably to those reported in similar studies predicting the synthesis of aerogels at pilot scale, as shown in Table 3, given the limited number of experiments available in the dataset, the model’s true predictive capability on unseen data might be overestimated, and its generalization to broader operating conditions may be constrained. The comparison with existing references is therefore intended as a contextual benchmark rather than as evidence of categorical superiority.

#### 2.3.3. Elastic Net Model for Thermal Conductivity

The same models as for the density were tested for predicting thermal conductivity (Table 5). Unfortunately, the results of the models were significantly worse than those for density, so the simplest model was chosen as a means of estimating the order of magnitude of the thermal conductivity for the minimum density point chosen.

The model errors (RMSE = 0.0036, MAE = 0.0027 W/m·K) are within the upper range of the variability observed between replicate or near-replicate experiments, which spans 0–0.004 W/m·K across the dataset. The relatively low R^2^ value is partly explained by the limited variance of thermal conductivity values resulting from the clustering of experiments within a narrow region of the design space.

The only relevant variable determined by the model is the ratio EDA/H12MDI.

## 3. Conclusions

The integration of a Digital Twin (DT) significantly enhanced process understanding by providing access to configured models and tools, such as dashboards and KPIs that enabled informed decision-making for optimizing both production and material properties. The developed Support Vector Regression (SVR) model provided high predictive accuracy (R^2^ = 0.964) for aerogel density and proved effective in guiding optimization of the synthesis pathway. Model-based exploration of experimental space revealed the operational windows most favorable for reducing density and improving thermal insulation. PEG molecular weights around 3000 g/mol, EDA/HMDI ratios between 0.15 and 0.25 mol/mol, and solids contents below 12 wt.% were identified as the most effective parameters for achieving minimal density while avoiding agglomeration or stability issues. Mechanical agitation was found to be the most suitable dispersion method for producing low-density aerogels. Although reducing solids content generally improved material properties, excessively low values compromised proper aerogel formation. Nevertheless, all these conclusions must be understood in the context of the limited dataset available and should be confirmed by further experiments before being applied beyond the explored design space.

Overall, this study demonstrates the value of combining experimental synthesis, predictive modelling, and DT technology to accelerate PU aerogel development, reduce reliance on trial-and-error experimentation and support pilot-scale implementation. Future efforts should focus on expanding the dataset and refining model features and strengthening the DT infrastructure to enable broader scalability and facilitate industrial deployment. By aligning data-driven engineering with sustainable manufacturing principles, this approach contributes to the development of smart materials and more environmentally responsible production systems.

## 4. Materials and Methods

### 4.1. Digital Twin Methodology

The web-based DT platform used in this research integrates a reference methodology [37] to support its implementation in the biomanufacturing sector, focusing on three key areas: data management, accessibility of DT tools, and knowledge management. This methodology was selected due to the lack of standardized approaches for deploying DTs in this sector.

Data management is a critical issue, as traditional practices often result in fragmented and inconsistent data stored in incompatible formats across inaccessible infrastructures. These challenges increase the complexity and cost of DT development [38]. The platform addresses this by standardizing data collection through a reference map of parameters that describe the process and link data to specific lifecycle stages, ensuring consistency and integration.

Accessibility of DT tools is another challenge, as bioprocess experts often lack data science expertise, limiting their ability to build, interpret, or improve DT models [38]. The platform simplifies this process by providing a user-friendly interface that allows users to manage models, whether developed within the platform or imported from external sources. It enables analyses such as what-if scenarios, optimizations, and feasibility studies, reducing reliance on real-world experimentation.

Finally, knowledge management is enhanced by integrating QbD and QbC principles [39]. QbD is supported through tools for designing and conducting experiments, while QbC is implemented via threshold-based notifications. These thresholds can be simple metrics like averages or advanced AI-driven triggers, thereby enabling precise monitoring and proactive adjustments.

**Figure 5:**
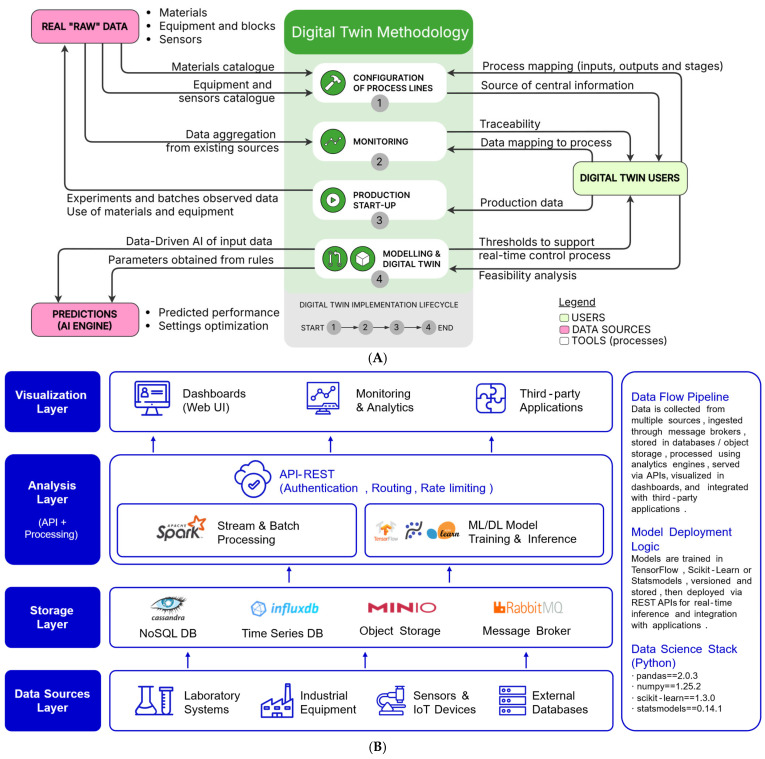
(**A**) Data flow and uses relationships between users, data sources and tools in the DT methodology. (**B**) Data pipeline and analytics architecture for DT implementation, including AI components.

The following sections detail how the platform addresses these challenges, focusing on its main components: process line configuration, monitoring and modelling, production start-up, and DT functionality. These are described in the context of their application to aerogel production, following a structured lifecycle process. Figure 5 provides an overview of both the system architecture and the data flow, illustrating the interactions between users, data sources, and tools within the DT framework.

#### 4.1.1. Configuration of Process Lines

The initial phase focused on identifying the primary data components: production process, materials, equipment, and sensors. A total of 81 parameters were mapped across different aerogel production stages to ensure comprehensive coverage of all variables that may affect the process and final material properties. From this initial set, six parameters were selected for the DT model based on their direct influence on density and thermal conductivity, as well as their consistency and availability across all experiments. Additionally, 13 materials and 12 pieces of equipment were catalogued to encompass all machinery involved in production and material characterization.

#### 4.1.2. Monitoring

The DT platform enabled data integration and traceability. Data was uploaded using standardized CSV templates, with registered equipment capable of integrating external data via REST API or OPC UA protocols, advancing the pilot plant’s digitalization. Traceability linked data samples to equipment, experiments, and batches, improving product identification and facilitating data sharing.

#### 4.1.3. Production Start-Up

Knowledge management was strengthened by linking observed data to the production context. Experimental data, derived from the mapped process parameters, included settings, material properties, and results. A total of 21 experiments were recorded in the DT, ensuring comprehensive traceability.

#### 4.1.4. Modelling and Digital Twin

The DT platform offered tools for modelling and interacting with simulations. Two types of models were supported: predefined models (e.g., ANOVA regressions, Support Vector Machines) and custom-developed models integrated from external sources. For this research, a Support Vector Regression (SVR) model was developed outside the platform to predict aerogel density, demonstrating its feasibility for optimizing production parameters. Thermal conductivity was validated using a linear regression. The platform provided tools to explore models, including single-run predictions, optimization analyses, and visual outputs like 2D and 3D response plots. Key metrics and training data were accessible for evaluation, supporting interpretation and informed decision-making.

##### Modelling Strategy and Mathematical Toolbox

To identify suitable models for integration into the DT platform, several modelling strategies were tested using the available dataset.

An initial exploratory analysis was conducted in this research to characterize the dataset. It relied on basic analysis of distribution plots to identify patterns and outliers and Principal Component Analysis, which was the main tool used to explore the variance structure, dominant feature contributions, and the degree of linearity in the relationship between the variables.

Then, to model the relationship between input parameters and the target aerogel properties, two techniques were chosen: Ordinary Least Squares linear regression (OLS) with different kinds of regularization (L1, L2 or both) to provide a baseline and a fast analysis and SVR:

- OLS is a basic statistical method used to model the linear relationship between a dependent variable and one or more independent variables by minimizing the sum of squared differences between the observed and predicted values [40]. To avoid overfitting, especially when dealing with smaller datasets, it is recommended to add regularization terms to the sum of squared differences loss function. Lasso regression (which implements L1 regularization) includes a penalty term proportional to the absolute value of the sum of coefficients. Ridge regression (which implements L2 regularization) includes a penalty term proportional to the squared sum of coefficients. Elastic Net regression combines both L1 and L2 regularization. These techniques help to avoid overfitting by addressing multicollinearity issues among input features.
- SVR estimates continuous variables while maximizing the margin between predictions and actual data. In the context of this research, an SVR with a radial basis function kernel was employed to handle non-linear relationships between the input parameters and the target properties. Regularization parameters controlled the margin’s flexibility, balancing error tolerance and precision [41]. Compared to other non-linear regressors such as tree-based ensembles or neural networks, SVR is particularly appropriate for very small datasets, as its formulation is grounded in structural risk minimization, which explicitly bounds generalization error rather than minimizing only the training loss [42]. In addition, SVR methods are well known for their ability to handle nonlinear relationships and high-dimensional input spaces, which are common in polymer science applications [43].

Additionally, the input data to the models was standardized to set the mean of each feature to 0 and the standard deviation to 1. Finally, the models were trained both on the features themselves, and on up to 3 principal components, as a means of reducing dimensionality.

The data analysis and modelling were implemented in Python 3.11.1, using standard data science libraries such as pandas, NumPy, Scikit-Learn, and Statsmodels (Figure 5B).

##### Evaluation Metrics and Validation Strategy

The model performance was evaluated using the following metrics [44]: Mean Squared Error (MSE) whose formula is provided in Equation (1); Root Mean Squared Error (RMSE), Equation (2); Mean Absolute Error (MAE), Equation (3); and Coefficient of determination or R-Squared (R^2^), following Equation (4).

(1) $$MSE=\frac{1}{N}\sum_{i=1}^{N}{\left|y_{i}-{ŷ}_{i}\right|}^{2}$$

(2) $$RMSE=\sqrt{\frac{1}{N}\sum_{i=1}^{N}{\left|y_{i}-{ŷ}_{i}\right|}^{2}}$$

(3) $$MAE=\frac{1}{N}\sum_{i=1}^{N}\left|y_{i}-{ŷ}_{i}\right|$$

(4) $$R^{2}=1-\frac{\sum_{i=1}^{N}{\left(y_{i}-{ŷ}_{i}\right)}^{2}}{\sum_{i=1}^{N}{\left(y_{i}-\bar{y}\right)}^{2}}$$

where $y_{i}$ is the actual value, ${ŷ}_{i}$ is the predicted value, $\bar{y}$ is the average of real values and N is the number of data points.

To select the appropriate model parameters, such as those controlling the amount of regularization, the Leave-One-Out Cross-Validation (LOOCV) [45] method was used. It works by training the model while leaving out one data point at a time, which is used as the test set, and using the remaining data as the training set. The validation ends once every point has been used, and the parameters that perform best on the test sets are selected [45]. While LOOCV is well-suited to small datasets in terms of efficient data use, it cannot eliminate the risk of overfitting due to the restricted size of the dataset. Even if model selection was deliberately restricted to simple, regularized formulations as indicated previously, the results of the models should be interpreted within the scope of the experimental conditions covered by the dataset.

This method is particularly suitable as it provides an estimate of how the model is likely to perform on unseen data when the dataset size is reduced and splitting the data into training and test datasets could compromise model training.

### 4.2. Polyurethane Aerogel Design and Development Based on Digital Twin

The synthesis of polyurethane (PU)-based aerogels followed a two-step process involving sequential polymerization and subsequent freeze-drying cycle at a pilot line scale, as previously outlined [29,35]. Initially, various organic solvents, such as acetone, acetonitrile (ACN), and ethyl acetate (EtOAc), or a combination thereof, were assessed. Afterwards, the concentration of solids was adjusted during the final synthesis stage, achieved by adding the appropriate volume of water to ensure complete polymer dispersion via mechanical agitation or sonication for PU dispersion preparation. This led to the production of aerogel samples with varying solids content ranging from 3.2 to 10.8 wt.%. Additionally, poly(ethylene glycol) (PEG) with different molecular weights ranging from 600 to 4000 g/mol was incorporated in the prepolymerization step.

After that, the freeze-drying process, comprising multiple stages (freezing, primary drying, and secondary drying), is initiated to remove water from the dispersion through sublimation, leading to the formation of the final aerogel. Specifically, the process involves freezing the mixture for 5.5 h at −40 °C, followed by a 60 h primary drying phase at 25 °C and 200 µbar. Finally, a 10 h secondary drying step is conducted at 40 °C. Throughout the freeze-drying process, the cooling or heating ramps required to reach the target temperatures last for 1 h. All experiments considered for the development of the mathematical model are shown in the Supplementary Material (Table S1).

#### 4.2.1. Characterization Equipment

The density of the synthesized aerogels was determined using a 3D scanner (REXCAN DS3 Silver, eQuality Tech Inc., Rochester Hills, MI, USA). This device generated a three-dimensional image of each sample through ezScan v 3.26 software, and the corresponding volume was calculated using Geometric Wrap v2021.2.2 software. Specifically, three square-shaped samples were taken from different regions of each synthesized aerogel to improve homogeneity of measurement. The mass of these samples was measured beforehand, and their density (ρ, g/cm^3^) was calculated by dividing the mass by the volume obtained from the 3D scan. Additionally, the thermal conductivity of the synthesized aerogels was obtained by conducting heat transfer measurements between two parallel plates (HFM 300, Linseis, Selb, Germany). Samples with dimensions of 30 × 30 cm were used for this analysis, which was performed at five intermediate temperatures ranging from 0 to 40 °C. Both density and thermal conductivity analyses were carried out in triplicate.

#### 4.2.2. DT Data Management

Data was manually collected by lab operators following a three-step procedure to ensure consistency. First, a map of the PU aerogel production process was created, identifying 81 parameters across 26 elements: 13 materials, 10 process stages, and 3 additional elements (unions, inspection stages, and final product parameters). Next, a structured dataset was built using a spreadsheet based on the process map, containing data from various experiments. Finally, the data was uploaded to the platform, linking it to 21 defined experiments to ensure traceability (Table S1).

#### 4.2.3. DT Interface

Users can access the DT’s application services (monitoring, simulation, optimization) via a web browser. The DT is implemented as a cloud-based service to bypass local hardware limitations, ensure consistency, and support collaboration through a centralized hub where model and process data are shared. For more details, see Supplementary Materials (Figures S1–S5).

## Figures and Tables

**Table 1:** Density and thermal conductivity results obtained for PU samples. Values are reported as mean ± standard deviation (SD) (*n* = 5).

PU Sample	Parameter	
	Density (g/cm^3^)	Thermal Conductivity (W/m·K)
PU-1	0.070 ± 0.001	0.042 ± 0.001
PU-2	0.121 ± 0.002	0.051 ± 0.002
PU-3	0.086 ± 0.002	0.045 ± 0.001
PU-4	0.051 ± 0.002	0.041 ± 0.001
PU-5	0.049 ± 0.003	0.037 ± 0.001
PU-6	0.051 ± 0.002	0.040 ± 0.001
PU-7	0.064 ± 0.002	0.034 ± 0.001
PU-8	0.051 ± 0.002	0.034 ± 0.001
PU-9	0.046 ± 0.002	0.032 ± 0.001
PU-10	0.117 ± 0.002	0.038 ± 0.002
PU-11	0.046 ± 0.002	0.031 ± 0.001
PU-12	0.059 ± 0.002	0.030 ± 0.001
PU-13	0.117 ± 0.003	0.038 ± 0.001
PU-14	0.048 ± 0.002	0.033 ± 0.001
PU-15	0.047 ± 0.002	0.033 ± 0.001
PU-16	0.050 ± 0.002	0.033 ± 0.001
PU-17	0.055 ± 0.003	0.034 ± 0.001
PU-18	0.052 ± 0.002	0.035 ± 0.001
PU-19	0.047 ± 0.002	0.037 ± 0.002
PU-20	0.034 ± 0.001	0.031 ± 0.001
PU-21	0.047 ± 0.002	0.033 ± 0.001

**Table 2:** Coefficients of the Elastic Net linear model for each input factor and the independent term.

Factor	Coefficient
EDA/H12MDI (mol/mol)	0
Sonication (binary)	0
PEG Molecular weight (g/mol)	0
Solids content (wt. %)	8.71 × 10^−3^
Independent term	2.09 × 10^−2^

**Table 3:** Summary of performance metrics obtained by the developed SVR and Elastic Net models and other authors. All values are reported to three decimal places for consistency; trailing zeros were added solely for formatting and do not indicate increased precision.

	Material type	Target Property	Dataset	Performance Metric			
Source				R^2^	MSE	RMSE	MAE
Hosseinpoor et al. RSM [26]	Carbon aerogel	Cefixime adsorption efficiency	31 experimental samples	0.750	8.800 × 10^−4^	2.900 × 10^−2^	n.a.
Hosseinpoor et al. ANN [26]	Carbon aerogel	Cefixime adsorption efficiency	31 experimental samples	0.800	6.900 × 10^−4^	2.600 × 10^−2^	n.a.
Zhou et al. ANN [27]	Silica aerogel glazing	Heat flux	Mathematical model-generated	0.740	n.a.	13.600	9.570
Zhou et al. ANN [27]	Silica aerogel glazing	Total heat gain	Mathematical model-generated	0.870	n.a.	11.200	8.020
Chao et al. SVM [36]	MXene/nanocellulose Hybrid Aerogel	Compressive modulus	34 experimental samples	0.867	4.070	2.020	n.a.
Chao et al. ANN [36]	MXene/nanocellulose Hybrid Aerogel	Compressive modulus	34 experimental samples	0.960	1.010	1.000	n.a.
This work SVR model	PUR aerogel	Density	21 experimental samples	0.964	2.300 × 10^−5^	4.800 × 10^−3^	4.200 × 10^−3^
This work Elastic Net model	PUR aerogel	Density	21 experimental samples	0.820	1.200 × 10^−4^	1.100 × 10^−2^	7.200 × 10^−3^

n.a.: not available.

**Table 4:** Hyperparameters of the SVR model.

Hyperparameter	Value
Kernel	RBF (Radial Basis Function)
C	0.1
ε	3.79 × 10^−3^

**Table 5:** Independent terms and coefficients of the Elastic Net linear model for predicting thermal conductivity for each input factor.

Factor	Coefficient
EDA/H12MDI (mol/mol)	3.57 × 10^−2^
Sonication (binary)	0
PEG Molecular weight (g/mol)	0
Solids content (wt. %)	0
Independent term	2.80 × 10^−2^

## Data Availability

The original contributions presented in this study are included in the article/Supplementary Material. Further inquiries can be directed to the corresponding author.

## Supplementary Materials

The following supporting information can be downloaded at: https://www.mdpi.com/article/10.3390/gels12060483/s1, Figure S1. Screenshot of the centralized access to model through Digital Twin. Created simulations (A) and details of the execution of one simulation (B). Figure S2. Screenshot of the interface to interact with existing model by typewriting the input values to run the model through a single execution (A) or an optimization (B). Figure S3. Screenshot of two different types of charts to explore models. Contour plot (A) and feasible region (B). Figure S4. Screenshot of the KPI sheet, in this example corresponding to density. It encompasses metadata information about the feature of interest, displays first and last values, the active alerts linked with the KPI and the time evolution of the observed values. Figure S5. Dashboard configured for pilot-scale PUR aerogel plant containing the values of the main KPIs and the most relevant parameters such as solids content, EDA/HMDI ratio or density. Table S1. Dataset of experiments considered for the study. N/A stands for “Not available”.

## Abbreviations

CAN	Acetonitrile
AI	Artificial Intelligence
ANOVA	Analysis of Variance
API REST	Application Programming Interface Representational State Transfer
CSV	Comma Separated Values
DMPA	Dimethylol propionic acid
DT	Digital Twin
EDA	Ethylenediamine
EtOAc	Ethyl acetate
HMDI	Hexamethylene Diisocyanate
KPI	Key Performance Indicator
MAE	Mean Absolute Error
ML	Machine Learning
MSE	Mean Squared Error
OPC UA	OLE (Object Linking and Embedding) for Process Control Unified Architecture
PEG	Poly(ethylene glycol)
PU	Polyurethane
QbC	Quality by Control
QbD	Quality by Design
QbDD	Quality by Digital Design
RMSE	Root Mean Squared Error
SVR	Support Vector Regression
SVM	Support Vector Machine
TEA	Triethylamine
