# Supplementary material for: Digital Twin-Driven Optimization of Pilot-Scale Polyurethane Aerogel Production Using SVR Modelling
Source: Gels. 2026 Jun 1;12(6):483. doi: 10.3390/gels12060483 (PMC13298227; doi:10.3390/gels12060483)
Supplement: Supplementary file 1 [file gels-12-00483-s001.zip › gels-4291232-supplementary.pdf]

Digital Twin-Driven Optimization of Pilot-Scale Polyurethane Aerogel Production using SVR Modelling

Óscar Brandón-Basdediós[1], Laura Miguélez-Riádigos[1], Esther Pinilla-Peñalver[2], Mateo Alonso[1], Paula Sánchez[2], Luz Sánchez-Silva[2]* and Juan Luis Sobreira-Seoane[1]

[1]Instituto Tecnológico de Galicia (ITG). Cantón Grande 9, Planta 3, 15003 A Coruña, Spain

[2]Department of Chemical Engineering, University of Castilla-La Mancha. Avda. Camilo José Cela 12, 13071 Ciudad Real, Spain

*Corresponding author e-mail: marialuz.sanchez@uclm.es, ORCID: 0000-0002-4348-7520

(A) (B)

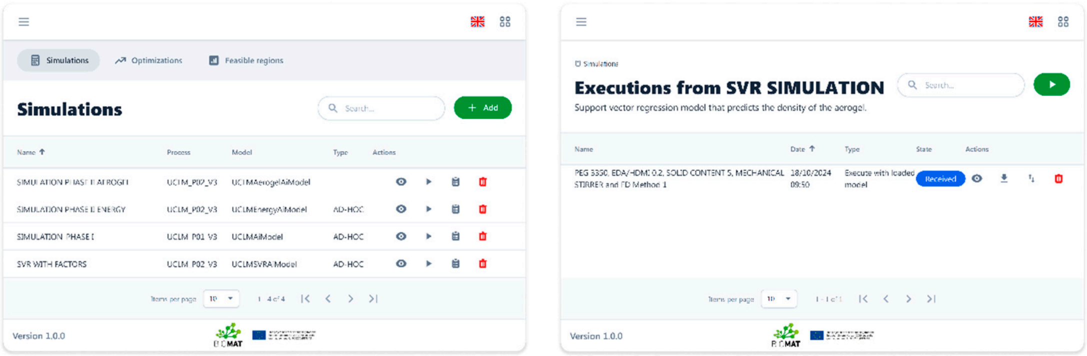

Figure S1. Screenshot of the centralized access to model through the Digital Twin. Created simulations (A) and details of the execution of one simulation (B).

(A) (B)

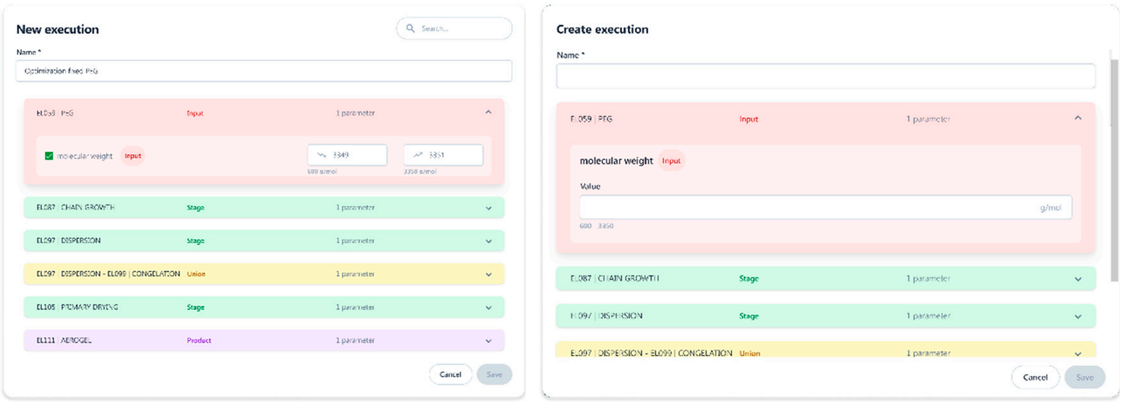

Figure S2. Screenshot of the interface to interact with existing model by typewriting the input values to run the model through a single execution (A) or an optimization (B).

(A) (B)

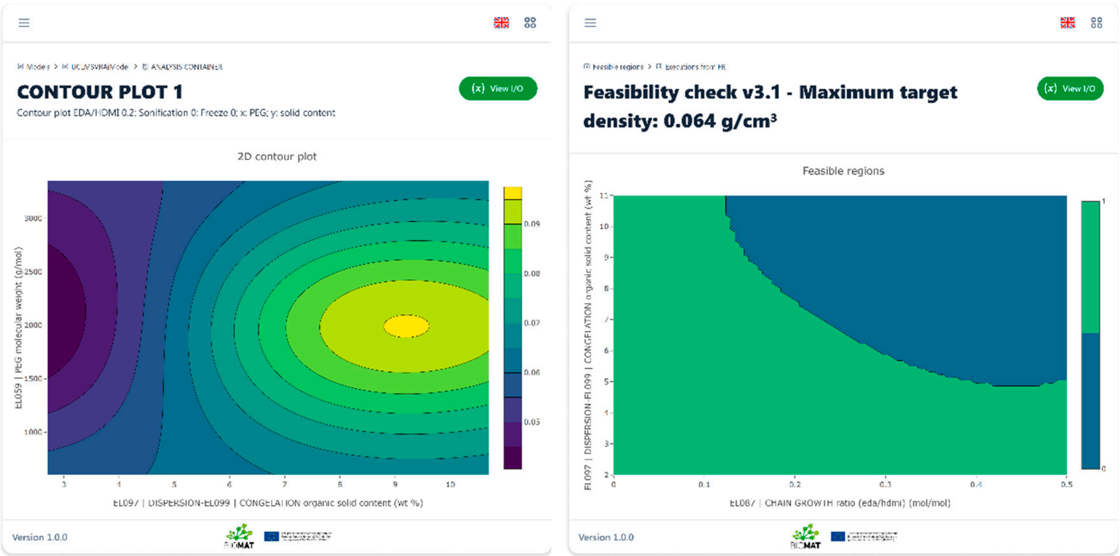

Figure S3. Screenshot of two different types of charts to explore models. Contour plot (A) and feasible region (B).

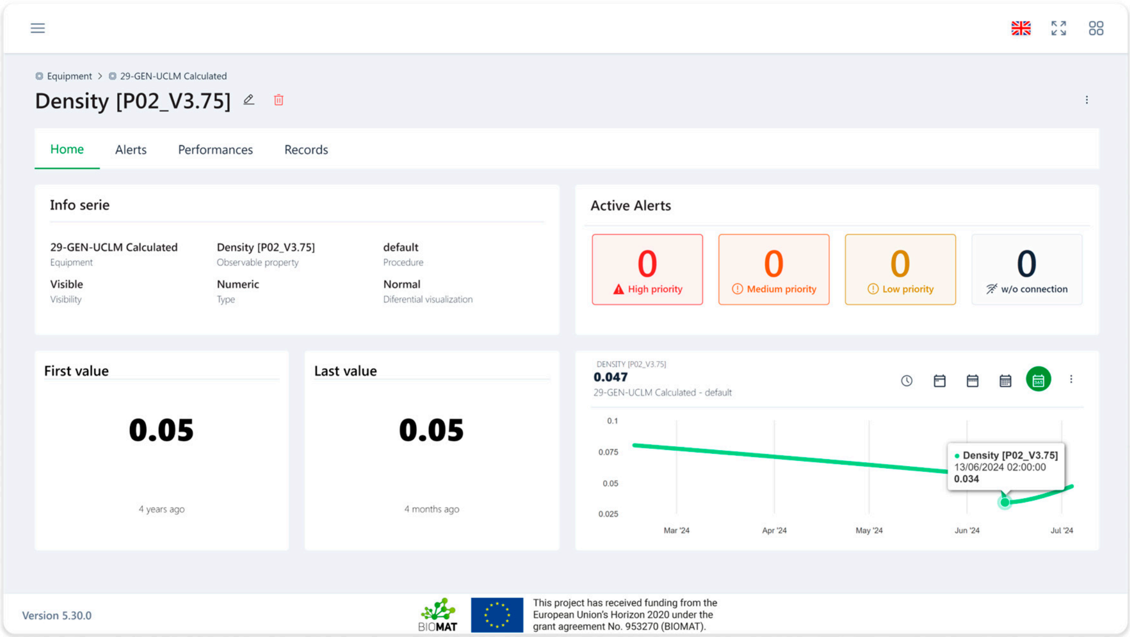

Figure S4. Screenshot of the KPI sheet, in this example corresponding to density. It encompasses metadata information about the feature of interest, displays first and last values, the active alerts linked with the KPI and the time evolution of the observed values.

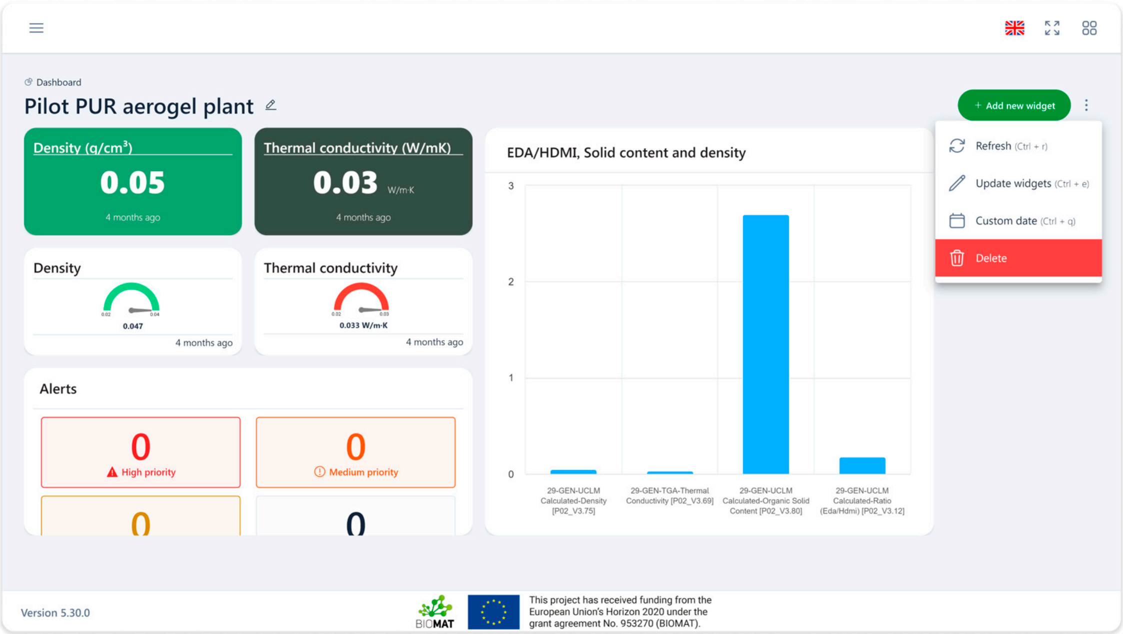

Figure S5. Dashboard configured for pilot-scale PUR aerogel plant containing the values of the main KPIs and the most relevant parameters such as solids content, EDA/HMDI ratio or density.

Table S1. Dataset of experiments considered for the study. N/A stands for "Not available"

	**Element**													
	INPUT: PEG	INPUT: PEG	INPUT: Ethyl acetate	INPUT: DMPA	INPUT: HMDI	INPUT: NMP	INPUT: DBTDL	INPUT: TEA	INPUT: EDA	INPUT: H2O	STAGE: Chain growth	STAGE: Dispersion	STAGE: Dispersion	UNION
	**Parameter**													
	Quantity	Molecular weight	Quantity	Quantity	Quantity	Quantity	Quantity	Quantity	Quantity	Quantity	Ratio (EDA/HMDI)	Mechanical stirring	Sonification stirring	Organic solids content
	**Units**													
**PUR sample**	g	g/mol	ml	g	ml	ml	ml	ml	ml	ml	mol/mol	rpm	kHz	wt %
PU-1	33.0	2000	116.1	6.6	24.3	4.8	0.2	8.2	2.2	904.8	0.334	N/A	450	7.50
PU-2	112.0	2000	394.7	22.4	82.5	16.2	0.5	28.0	7.5	3196.7	0.334	900	N/A	7.20
PU-3	112.0	2000	394.7	22.4	82.5	16.2	0.5	28.0	7.5	3196.7	0.334	N/A	450	7.20
PU-4	112.0	2000	394.7	22.4	82.5	16.2	0.5	28.0	7.5	7795.0	0.334	900	N/A	3.10
PU-5	112.0	2000	394.7	22.4	82.5	16.2	0.5	28.0	7.5	6561.7	0.334	900	N/A	3.70
PU-6	112.0	2000	394.7	22.4	82.5	16.2	0.5	28.0	7.5	6561.7	0.334	N/A	450	3.70
PU-7	112.0	2000	394.7	22.5	82.5	16.2	0.5	28.0	2.0	6555.4	0.090	900	N/A	3.60
PU-8	112.0	2000	394.7	22.5	82.5	16.2	0.5	28.0	3.0	6590.7	0.130	900	N/A	3.60
PU-9	112.0	2000	394.7	22.5	82.5	16.2	0.5	28.0	4.0	6626.0	0.180	900	N/A	3.60
PU-10	112.0	2000	394.7	22.4	82.5	16.2	0.5	28.0	7.5	2075.1	0.334	900	N/A	10.70
PU-11	112.0	2000	394.7	22.5	82.5	16.2	0.5	28.0	4.0	6626.0	0.180	N/A	450	3.60
PU-12	112.0	2000	394.7	22.5	82.5	16.2	0.5	28.0	4.0	6626.0	0.180	N/A	450	3.60
PU-13	112.0	2000	394.7	22.4	82.5	16.2	0.5	28.0	7.5	2075.1	0.334	900	N/A	10.70
PU-14	112.0	2000	394.7	22.5	82.5	16.2	0.5	28.0	5.0	6661.4	0.220	900	N/A	3.60
PU-15	112.0	2000	394.7	22.5	82.5	16.2	0.5	28.0	5.0	6661.4	0.220	N/A	450	3.60
PU-16	112.0	2000	394.7	22.5	82.5	16.2	0.5	28.0	3.0	6590.7	0.130	900	N/A	3.60
PU-17	33.6	600	394.7	22.5	82.5	16.2	0.5	28.0	4.0	3570.0	0.180	900	N/A	4.50
PU-18	56.0	1000	394.7	22.5	82.5	16.2	0.5	28.0	4.0	4444.0	0.180	900	N/A	4.10

	Element													
	INPUT: PEG	INPUT: PEG	INPUT: Ethyl acetate	INPUT: DMPA	INPUT: HMDI	INPUT: NMP	INPUT: DBTDL	INPUT: TEA	INPUT: EDA	INPUT: H2O	STAGE: Chain growth	STAGE: Dispersion	STAGE: Dispersion	UNION
	**Parameter**													
	Quantity	Molecular weight	Quantity	Quantity	Quantity	Quantity	Quantity	Quantity	Quantity	Quantity	Ratio (EDA/HMDI)	Mechanical stirring	Sonification stirring	Organic solids content
	**Units**													
**PUR sample**	g	g/mol	ml	g	ml	ml	ml	ml	ml	ml	mol/mol	rpm	kHz	wt %
PU-19	112.0	2000	394.7	22.5	82.5	16.2	0.5	28.0	4.0	8888.0	0.180	900	N/A	2.70
PU-20	188.0	3350	394.7	22.5	82.5	16.2	0.5	28.0	4.0	9576.0	0.180	900	N/A	3.30
PU-21	112.0	2000	394.7	22.5	82.5	16.2	0.5	28.0	4.0	8888.0	0.180	900	N/A	2.70
